# Cystic versus non-cystic silent corticotrophic adenomas: clinical and histological analysis of 62 cases after microscopic transsphenoidal surgery—a retrospective, single-center study

**DOI:** 10.1038/s41598-023-29628-3

**Published:** 2023-02-11

**Authors:** Piotr Sumislawski, Torge Huckhagel, Kara Leigh Krajewski, Jens Aberle, Wolfgang Saeger, Jörg Flitsch, Roman Rotermund

**Affiliations:** 1 Department of Neurosurgery, University Medical Center Hamburg-Eppendorf, Martinistr. 52, 20246 Hamburg, Germany; 2 Department of Pediatric Neurosurgery, AKK Altona Children’s Hospital, Bleickenallee 38, 22763 Hamburg, Germany; 3 Department of Medicine, University Medical Center Hamburg-Eppendorf, Hamburg, Germany; 4 Institute of Neuropathology, University Medical Center Hamburg-Eppendorf, Hamburg, Germany; 5 Department of Neuroradiology, University Clinic Göttingen, Göttingen, Germany; 6 Department of Neurosurgery, University clinic Carl Gustav Carus, Technische Universität Dresden, Dresden, Germany; 7 Department of Neurosurgery, Diako Krankenhaus Flensburg, Flensburg, Germany

**Keywords:** Surgical oncology, Endocrine system and metabolic diseases

## Abstract

Silent corticotrophic adenomas (SCAs) represent a rare group of non-functioning adenomas with a potentially aggressive clinical course. Cystic component is a very common finding among SCAs, but its clinical relevance has not yet been investigated. The aim of this study was to analyze clinical features of cystic and non-cystic SCAs, perioperative complications after microscopic transsphenoidal surgery, clinical outcome after first and repeat surgery along with risk factors for recurrence. We conducted a retrospective analysis of 62 silent corticotrophic adenomas treated at our university medical center via microscopic transsphenoidal surgery between January 2008 and July 2019. Parameters investigated included histology, invasiveness, intratumoral haemorrhage or cystic component on MRI, perioperative alteration of visual field, tumor size, pre- and postoperative ACTH, FSH, GH, LH, TSH, prolactin, cortisol, free T4, free T3, IGF-1, estrogen and testosterone levels, perioperative complications, neoadjuvant and adjuvant therapy along with clinical outcomes. A total of 62 patients were analyzed. The mean follow up was 28.3 months. Tumors with a cystic component occur statistically significant more often among male than non-cystic (80.6% vs. 44.4%, *p* = 0.02) and display lower rates of cavernous sinus invasion and sphenoid sinus invasion were significantly lower for cystic lesions comparing to non-cystic tumors (42.3% vs. 69.4%, *p* = 0.04 and 3.8% vs. 47.2%, *p* < 0.001). GTR after MTS was not statistically significant higher by cystic SCAs (80% vs. 57.1%, *p* = 0.09). Cystic lesions were also associated with higher risk of hyperprolactinemia (19.4% vs. 2.8%, *p* = 0.02) and only densely granulated cystic SCAs presented with preoperative intratumoral hemorrhage (19.2% vs. 0%, *p* = 0.01). Mean duration of first surgery was significantly shorter for cystic SCAs (71.6(± 18.7) vs. 94.8(± 31.1) minutes, *p* = 0.01). Preoperative pituitary insufficiency (25% vs. 16.7%, *p* = 0.49), intraoperative CSF space opening (21.1% vs. 37.5%, *p* = 0.32), along with postoperative new pituitary insufficiency (15% vs. 10%, *p* = 0.67) or diabetes insipidus/SIADH (10% vs. 13.3%, *p* > 0.99) with histological markers such as Ki67 (21.1% vs. 13.8%, *p* = 0.70) and p53 expression (6.3% vs. 0%, *p* = 0.39) as well as mitotic rate (5.3% vs. 10.3%, *p* > 0.99) were comparable between both groups. The presence of cystic component did not affect the tumor recurrence (10% vs. 16%, *p* = 0.68). Mean duration of surgery was first surgeries was not statistically shorter than repeat surgeries (85.4 ± 29.1 vs. 93.8 ± 28 min, *p* = 0.15). Patients undergoing first surgery had a higher probability of gross total resection (74.4% vs. 30%, *p* = 0.01) and lower probability of intraoperative CSF space opening (26% vs. 58.3%, *p* = 0.04) as well as a lower rate of preoperative anterior pituitary insufficiency (20% vs. 58.3%, *p* = 0.01). The incidence of new postoperative anterior pituitary insufficiency (10% vs. 0%, *p* = 0.57) and transient diabetes insipidus/SIADH (12% vs. 8.3%, *p* > 0.99) between those groups were comparable. No statistical difference was observed between patients with remission and with recurrent tumor regarding cortisol and ACTH levels, incidence of different histological subgroups, invasively growing tumors and lesions with cystic components as well as the percentage of cases with increased Ki67 proliferation index, p53 expression and mitotic indices. Our study presents one of the largest available cohorts of SCAs after microscopic transsphenoidal surgery and first clinical analysis of cystic versus non-cystic SCAs so far. We also performed the first comparison of index and repeat surgeries for this tumor entity. Cystic tumors presented with characteristic clinical aspects like male predominance, higher risk of hyperprolactinemia as well as lower rates of cavernous sinus and sphenoid sinus invasion comparing to non-cystic lesions. Mean duration of first surgery was significantly shorter for cystic SCAs. Moreover preoperative intratumoral hemorrhage had 100% specificity and 60% sensitivity for densely granulated cystic SCAs. All these clinical hallmarks may suggest a novel subgroup of SCAs with distinct clinical and biological features, however further clinical and molecular investigations are required. Second surgeries are associated with a higher incidence of preoperative pituitary insufficiency, and a higher risk of subtotal resection, and a higher probability of CSF space opening intraoperatively compared to first surgeries. On the other hand, the risk of new postoperative pituitary insufficiency was higher after first surgeries. In our cohort of patients, no prognostic factor for recurrence among histological diagnosis, Ki67-proliferation index, p53 expression, number of mitoses, invasive growth or cystic lesions for SCAs could be detected.

## Introduction

Silent corticotrophic adenomas (SCAs) are a group of rare, non-functioning adenomas comprising 3–6% of all pituitary adenomas and up to 20% of corticotrophic adenomas^1,2^. Their prevalence among non-functioning adenomas (NFAs) is 5.5%^3^. SCAs are hormonally inactive, which may be the reason they are diagnosed at an advanced stage of the disease usually as macroadenomas and manifesting the corresponding “mass effect” with visual impairment or cranial nerve defect as a first sign, which is in contrast to hormonally active adenomas^4^.

According to current WHO 2017 classification, the diagnosis of the corticotrophic adenoma is defined as Tpit and ACTH positive or negative pituitary tumor with no clinical sign of hypercortisolism. The prevalence of ACTH positive and ACTH negative silent corticotrope adenoma is inconsistent between the studies with 37.1—61.4% and 48.6—62.9% of tumors respectively^5,6^. Based on granulation and the presence of Crooke cells, corticotrophic adenomas can be further divided into three groups: densely granulated corticotrophic adenoma, sparsely granulated corticotrophic adenoma and Crooke cell adenoma^7^. SCAs can be classified either as type 1 SCA (densely granulated) or type 2 SCA (sparsely granulated)^6^. Silent Crooke cell adenoma have also been reported^8^. The incidence of type 2 SCA is higher than type 1 SCA and associated with a lower expression of POMC and PC1/3 enzyme, which may explain why type 1 SCAs more frequently develop into Cushing`s disease than type 2 SCA^9–11^. Reverse transformation from Cushing’s disease into SCAs has also been reported^12^. Cystic component is a very common finding among SCAs as compared to other NFAs^2,13,14^, but its clinical relevance has not yet been investigated. Two different types of cystic components: microcysts (< 3 mm) and macrocysts within the tumors have been described in the literature and may be helpful to distinguish SCAs from other NFAs^2,14,15^. Important differential diagnoses for cystic pituitary adenoma remain craniopharyngioma, Rathke's cleft cyst, colloid cyst, arachnoid cyst, epidermoid cyst or abscess^16^.

SCAs were also described to be more aggressive than other NFAs as they exhibit higher sinus cavernosus invasion and recurrence rates^1^. To assess the prognosis of pituitary adenoma according to current WHO 2017 classification different clinical factors such as tumor size (microadenoma, macroadenoma or giant tumor), clinical presentation (clinically functioning, clinically non-functioning, or silent), invasiveness both intraoperatively and on MRI as well as histological markers like Ki-67 proliferation index, p53 expression along with the mitotic index are taken into account^17,18^. Ki-67 identifies the cells in late G1, S, G2, and M phases^19^. PH3 staining for mitotic index reveals cells only in the M phase^20^. Increased p53 expression may be an indicator for p53 mutation^21^. Cut-off values among these markers significant for more aggressive tumors are Ki-67 proliferation index ≥ 3%, p53 expression ≥ 2% and ≥ 2 mitoses per 10HPFs^22^.

The aim of this study was to analyze clinical features of cystic and non-cystic SCAs, perioperative complications after microscopic transsphenoidal surgery, clinical outcome after first and repeat surgery along with risk factors for recurrence.

## Methods

### Patient selection

We retrospectively reviewed the medical records of 62 silent corticotrophic adenomas treated at our university medical center via microscopic transsphenoidal surgery between January 2008 and July 2019. The inclusion criteria were: evidence of pituitary tumor on the preoperative MRI, clinically and endocrinologically diagnosed as non-functioning pituitary adenoma (NFPA) without hypercortisolemic/cushingoid features and positive Tpit staining with positive or negative ACTH immunoreactivity.

### Laboratory studies

Serum cortisol, adreno-corticotropic hormone (ACTH), prolactin (PRL), follicle-stimulating hormone (FSH), luteinizing hormone (LH), testosterone or estrogen, growth hormone (GH), insulin-like growth factor-1 (IGF-1), thyroid-stimulating hormone (TSH), and thyroid hormones (fT3 and fT4) were examined preoperatively one day before surgery and were drawn postoperatively on the first and third postoperative days as well as on varying postoperative days before discharge. Complete hormone deficiency was defined as insufficient production of all anterior pituitary hormons, whereas by partial hormone deficiency minimum one pituitary axis was intact.

### Radiological evaluation

MRI were performed either in an outpatient setting or inpatient prior to surgery. Tumor size was measured on the preoperative MRI and then classified as micro- (< 1 cm), macroadenoma (≥ 1 cm and < 4 cm) or giant tumor (≥ 4 cm). Invasiveness including suprasellar, cavernous sinus, sphenoid sinus and clival invasion were additionally evaluated. Preoperative intratumoral bleeding seen on MRI and cystic components on T2 weighted images were also noted.

### Histological examination

Intraoperative specimens were fixed in 4% paraformaldehyde, dehydrated, embedded in paraffin and then sectioned at 4 µm according to standard lab protocols and underwent H & E staining as well as periodic acid–Schiff reaction staining. Immunohistochemistry for pituitary hormones (adreno-corticotropic hormone, somatotropic hormone, prolactin, follicle-stimulating hormone, luteinizing hormone, thyroid-stimulating hormone); S100 protein; pancytokeratin (KL1 or Ca m 5.2); Tpit expression; mitotic marker phosphohistone-3 (PH3); proliferation marker Ki-67 (MIB-1); and accumulation of tumor suppressor protein p53 were performed using an automated staining machine (Ventana BenchMark TX, Roche Diagnostics, Mannheim, Germany).

### Surgical procedure

All patients were operated via microscopic transsphenoidal surgery. The operative technique in a semisitting position with head rotated toward the surgeon has been previously described^23,24^. Duration of the operation was defined as incision-suture time.

### Follow-up

Remission of SCAs was defined as no residual tumor on the first postoperative MRI with no progression on following MRIs whereas recurrence was determined by presence of tumor progression on follow-up MRI.

### Statistical analyses

The data were acquired from the patients’ electronic files by systematic data search. Data are reported as means with standard deviations (SD) for continuous variables normally or not normally distributed, and as frequencies for categorical variables. Normal distribution was tested using the Kolmogorov–Smirnov test. Means were compared using the unpaired t-test when data distribution was normal, or by the Wilcoxon rank-sum test when variables were not normally distributed. For categorical analysis, a chi square test and Fisher’s exact test were used. P value < 0.05 was considered statistically significant. The statistical tests and data visualization were performed in GraphPad Prism (Version 9.3.0).

### Ethics statement

Approval of the study was obtained by the local ethics committee (Ethikkommission der Ärztekammer Hamburg). Informed consent was obtained from all patients (above 16 years old) and their legal guardian(s) (below 16 years of age). The study was performed in accordance with the Declaration of Helsinki.

## Results

### Demographic and clinical data

A total number of 62 patients with silent corticotrophic adenomas who underwent transsphenoidal surgery at our university medical center between January 2008 and July 2019 were analyzed. There were 26 females and 36 males in the cohort. Cystic lesions were observed in 26 cases (20 first and 6 repeated surgeries) with mean age of 57.6 years (range 15.3–78.6 years). Tumor size measurements performed retrospectively for these lesions per preoperative MRI revealed one microadenoma, 23 macroadenomas and 1 giant tumors with a mean tumor size 1.88 cm (coronal), 1.88 cm (sagittal) and 1.99 cm (axial). In one case, retrospective acquisition of data was not possible. On the other hand non-cystic lesion were identified among 36 patients (30 first and 6 repeated surgeries) with mean age of 54.1 years (range 22.8–83.8 years). Lesions were classified as 33 macroadenoma and 3 giant tumors based on diameter measurements with a mean tumor size 2.34 cm (coronal), 2.20 cm (sagittal) and 2.24 cm (axial).

Cystic SCAs tends to occur more often by male comparing to non-cystic tumors (80.6% vs. 44.4%, *p* = 0.02). Preoperative intratumoral bleeding was observed on five MRIs from cystic lesions. Regarding associated hyperprolactinemia in six specimens by cystic lesion and one among non-cystic tumors, no other causes could be identified. Preoperative ophthalmological examination displayed deterioration of visual fields by 32 patients equally distributed between cystic and non-cystic SCAs. Different vectors of tumor invasion were analyzed and revealed similar rate of suprasellar and clival invasion for both SCAs subgroups. Cavernous sinus invasion and sphenoid sinus invasion rates were significantly lower for cystic lesions comparing to non-cystic tumors. All data are shown in Table 1.

**Table 1:** Characteristics of 62 patients with cystic and non-cystic silent corticotrophic adenoma.

Characteristic	Cystic	Non-cystic	*p* value
Female	6 (19.4%)	20 (55.6%)	0.02
Age in yrs(range)	57.6 (15.3–78.6)	54.1 (22.8–83.8)	0.50
Duration of follow-up in months(range)	26.7 (1–73)	31.1 (3–95)	0.34
Tumor size			
Microadenomas	1 (3.8%)	0 (0%)	0.42
Macroadenomas	23 (88.6%)	33 (91.7%)	0.69
Giant tumors	1 (3.8%)	3 (8.3%)	0.63
No size available	1 (3.8%)	0 (0%)	0.42
Mean tumor size in cm cor*	1.88	2.34	0.08
Mean tumor size in cm sag*	1.88	2.20	0.95
Mean tumor size in cm ax*	1.99	2.24	> 0.99
Preoperative intratumoral hemorrhage	5 (19.2%)	0 (0%)	0.01
Associated hyperprolactinemia with prolactin levels < 100 ng/ml	6 (19.4%)	1 (2.8%)	0.02
Preoperative deterioration of visual fields	16 (61.5%)	16 (44.4%)	0.21
Invasive	21 (80.8%)	34 (94.4%)	0.12
Suprasellar invasion	16 (61.5%)	22 (61.1%)	> 0.99
Cavernous sinus invasion	11 (42.3%)	25 (69.4%)	0.04
Sphenoid sinus invasion	1 (3.8%)	17 (47.2%)	< 0.001
Clival invasion	1 (3.8%)	2 (5.6%)	> 0.99
First surgery	20 (80.6%)	30 (83.3%)	0.54
Repeat surgery	6 (19.4%)*	6 (16.7%)**	0.54
GTR	20/25 (80%)	16/28 (57.1%)	0.09

ax = axial, cm = centimeter; cor = coronal; sag = sagittal; yrs = years.

*4 patients—1 previous surgery, 1 patient—two previous surgeries, 1 patient—three previous surgeries; **3 patients—1 previous surgery, 2 patients—two previous surgeries, 1 patient—three previous surgeries.

### Histological analyses

All 62 consecutive cases were diagnosed as corticotrophic adenomas and were classified according to the WHO 2017 Classification of Tumours of Endocrine Organs. Crooke cell adenoma, densely granulated corticotrope adenomas and sparsely granulated corticotrope adenomas were observed in 1, 19 and 42 specimens, respectively. In our cohort all cases were ACTH positive and Tpit positive. All results are shown in Table 2.

**Table 2:** Histopathological subgroups according to the WHO 2017 classification.

Histological subgroups	No. of cases	%
Densely granulated corticotrophic adenoma	19	30.6
Sparsely granulated corticotrophic adenoma	42	67.7
Crooke’s cell adenoma	1	1.6

### Complications and distinctive features

The most common intraoperative feature was CSF space opening, which occurred in 20 of the cases analyzed (all with suprasellar invasion) and which was repaired with vastus lateralis muscle in 14, fat graft in two and only TachoSeal® with DuraSeal® in four cases, respectively. One surgery was accompanied by intraoperative bradycardia due to manipulation of the internal carotid artery. In the early postoperative phase before discharge, new partial pituitary insufficiency observed in 5 patients was the most frequent complication followed by three cases of diabetes insipidus. A later follow-up manifested sinusitis in three cases as well as two cases of diabetes insipidus. Other, less common complications are summarized in Table 3.

**Table 3:** Complications and surgical features after microscopic transsphenoidal surgery.

Complications and Surgical Features	Comment	%
Intraoperative		
20 CSF space opening	Reconstruction: 4 TachoSeal® with DuraSeal® only, 2 fat graft and 14 vastus lateralis muscle	32.3
1 Bradycardia	Only transient	1.6
Directly postoperatively		
5 partial pituitary insufficiency	(2 corticotrope,1 gonadotrope, 1 thyrotrope and 1 thyro-, gonado- and corticotrope)	8.1
3 diabetes insipidus	2 transient, 1 persistent	4.8
1 SIADH	only transient	1.6
1 CN palsy w/ diplopia	CN VI only transient	1.6
1 CSF-leakage		1.6
1 meningitis		1.6
1 postoperative bleeding	1 hematoma in the resection cavity	1.6
2 needed intensive care	Due to electrolyte imbalance	3.2
At later follow-up		
2 diabetes insipidus	1 persistent since surgery, 1 transient	3.2
1 SIADH	Only transient	1.6
1 CSF-leakage with meningitis		1.6
1 required intensive care	Due to SIADH	1.5
Other	1 renal insufficiency, 1 stroke of the recurrent artery of Heubner, 1 hypertensive crisis, 2 sinusitis	8.1

### Anterior pituitary insufficiency

**Figure 1:**
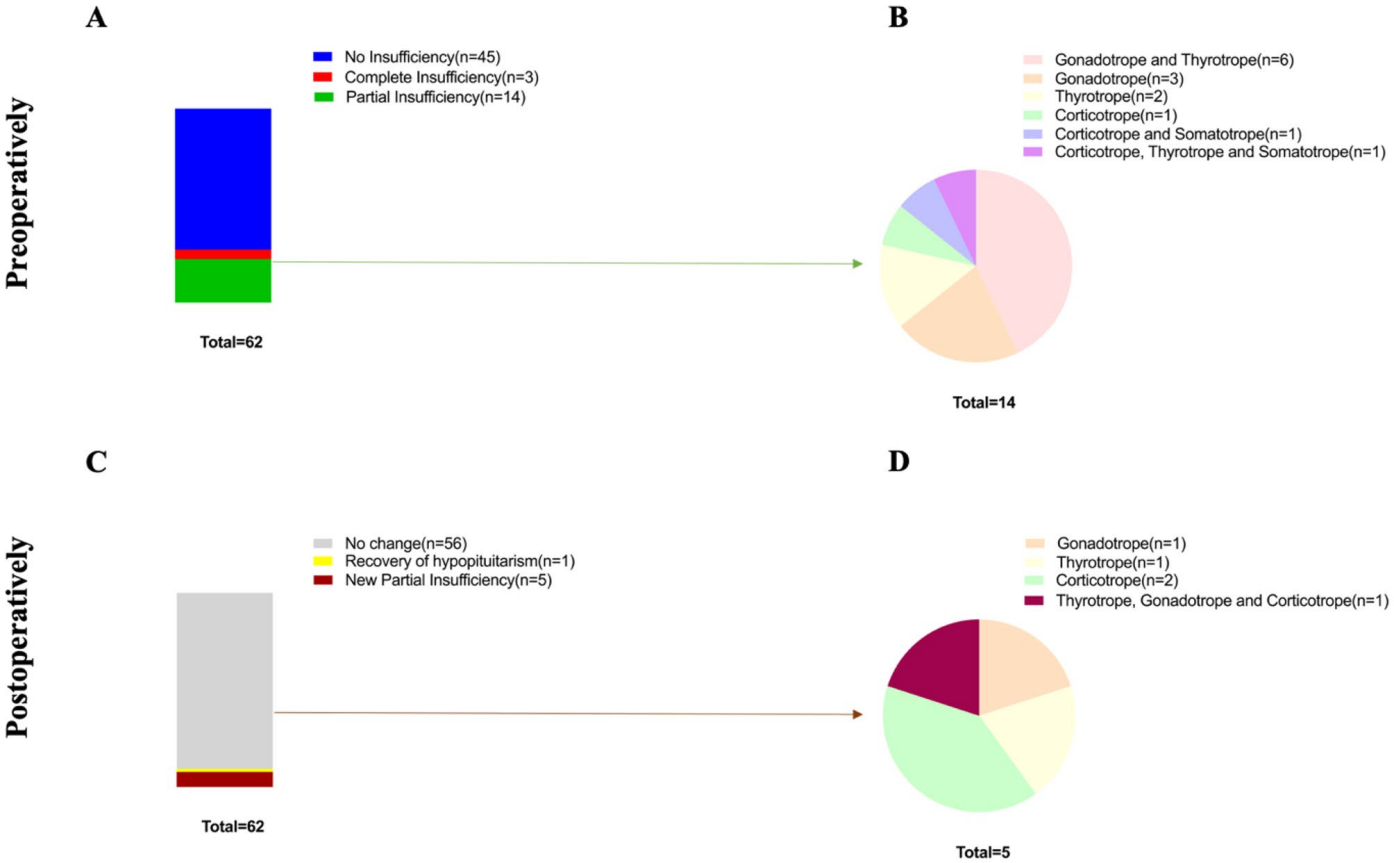
Preoperative and postoperative pituitary insufficiency. Pituitary insufficiency preoperatively (**A**,**B**) and postoperatively (**C**,**D**). Preoperative hypopituitarism was defined either as complete or partial insufficiency (**A**). Postoperative pituitary hormone deficiency was classified as no change, recovery from hypopituitarism or new partial pituitary insufficiency (**C**). Both preoperative and postoperative partial pituitary insufficiency were further characterized by type of hormone deficiency (**B**,**D**).

Preoperative laboratory examinations revealed three patients with complete and 14 patients with partial pituitary insufficiency (Fig. 1A). Partial insufficiency predominantly affected the gonadotropic axis followed by the thyreotropic axis (Fig. 1B). Postoperatively, one recovery from hypopituitarism and 5 new partial pituitary insufficiencies were observed (Fig. 1C,D). One postoperative corticotrope insufficiency may be explained by preoperative dexamethasone therapy before surgery.

### Follow-up and adjuvant treatment

All patients were invited for regular postoperative follow-up exams, which comprise clinical, laboratory and MRI evaluation. The mean follow up was 26.7 months (range 1–73 months) for cystic and 31.1 months (range 3–95 months) for non-cystic tumors (for both groups 28.3 months). Nine patients did not attend the recommended postoperative examination at our clinic. On follow-up MRI, gross total resection (GTR) was achieved in 36 cases (20 cystic lesions and 16 non-cystic SCAs) and was not statistically significant higher by cystic SCAs (80% vs. 57.1%, *p* = 0.09). Among 17 cases with primary STR (subtotal resection), 9 patients (5 with non-cystic and 4 with cystic lesions) presented with progression. One patient with non-cystic SCAs presented with recurrence after GTR. The remaining eight patients with residual tumor (4 non-cystic and 4 cystic) were regularly evaluated without any signs of progression. Eight out of 17 patients (4 non-cystic and 4 cystic) after STR were referred to radiotherapy. Follow-up was available for seven (87.5%) of those patients: seven patients underwent stereotactic radiotherapy (4 non-cystic and 3 cystic). Only four of them (2 cystic and 2 non-cystic tumors) reported remission on further follow-up. Two patients (1 non-cystic and 1 cystic lesion) received additionally cabergoline and they reported remission on further follow-up. One patient with cystic SCA was treated only with cabergoline and pasireotide, however without any success and was referred for two further surgeries.

### Primary surgery of cystic versus non-cystic silent corticotrope adenoma

From the patients who underwent first surgery we identified 20 cystic and 30 non-cystic SCAs. Analysis of preoperative MRI by tumors with and without cystic component revealed a statistically significant higher incidence of intratumoral hemorrhage (25% vs. 0%, *p* = 0.01) and hyperprolactinemia (30% vs. 3.3%, *p* = 0.01). Mean duration of surgery was significantly shorter for cystic SCAs (71.6(± 18.7) vs. 94.8(± 31.1) minutes, *p* = 0.01). GTR was amenable more frequent in cystic lesions (85% vs. 60%, *p* = 0.10), however without statistical significance. Preoperative pituitary insufficiency (25% vs. 16.7%, *p* = 0.49), intraoperative CSF space opening (21.1% vs. 37.5%, *p* = 0.32), along with postoperative new pituitary insufficiency (15% vs. 10%, *p* = 0.67) or diabetes insipidus/SIADH (10% vs. 13.3%, *p* > 0.99) with histological markers such as expression of Ki67 (21.1% vs. 13.8%, *p* = 0.70) and p53 (6.3% vs. 0%, *p* = 0.39) as well as mitotic rate (5.3% vs. 10.3%, *p* > 0.99) were comparable between both groups. The presence of cystic component did not affect the tumor recurrence (10% vs. 16%, *p* = 0.68). The complete comparison was summarized in Table 4.

**Table 4:** Comparison of clinical features of primary operated cystic and non-cystic silent corticotrophic adenomas.

Criteria	Cystic tumors (n = 20)	Non-cystic tumors (n = 30)	*p* value
Associated hyperprolactinemia with prolactin levels < 100 ng/ml	6/20 (30%)	1/30 (3.3%)	0.01
Preoperative intratumoral hemorrhage	5/20 (25%)*	0/30 (0%)	0.01
Mean(± SD) duration of surgery in minutes	71.6 (± 18.7)	94.8 (± 31.1)	0.01
GTR	17/20 (85%)	15/25 (60%)	0.10
Preoperative pituitary insufficiency	5/20 (25%)	5/30 (16.7%)	0.49
CSF space opening intraoperatively	4/19 (21.1%)	9/24 (37.5%)	0.32
Postoperative complications			
New pituitary insufficiency	3/20 (15%)	3/30 (10%)	0.67
SIADH or Diabetes insipidus	2/20 (10%)	4/30 (13.3%)	> 0.99
Histological markers			
Ki-67 ≥ 3%	4/19 (21.1%)	4/29 (13.8%)	0.70
P53 expression ≥ 2%	1/16 (6.3%)	0/25 (0%)	0.39
≥ 2 mitoses per 10 HPFs	1/19 (5.3%)	3/29 (10.3%)	> 0.99
Recurrence rate	2/20 (10%)	4/25 (16%)	0.68

*All hemorhages were present by densely granulated SCAs.

### Primary surgery versus repeat surgery

Mean duration of surgery was first surgeries was not statistically shorter than repeat surgeries (85.4 ± 29.1 vs. 93.8 ± 28 min, *p* = 0.15). GTR could be achieved more frequently by first surgeries (74.4% vs. 30%, *p* = 0.01). The risk of intraoperative CSF space opening was significantly lower in first surgeries (26% vs. 58.3%, *p* = 0.04). Preoperative pituitary insufficiency was observed statistically less commonly before first surgery (20% vs. 58.3%, *p* = 0.01). The risk of new postoperative pituitary insufficiency (10% vs. 0%, *p* = 0.57) and the incidence of diabetes insipidus/SIADH (12% vs. 8.3%, *p* > 0.99) was similar between these groups.

All results are shown in Table 5.

**Table 5:** Comparison of first surgeries versus repeat surgeries.

Criteria	First surgery (n = 50)	Repeat surgery (n = 12)	*p* value
Mean(± SD) duration of surgery in minutes	85.4 (± 29.1)	93.8 (± 28)	0.15
GTR	33/43 (76.7%)	3/10 (30%)	0.01
Preoperative pituitary insufficiency	10/50 (20%)	7/12 (58.3%)	0.01
CSF space opening intraoperatively	13/50 (26%)	7/12 (58.3%)	0.04
Postoperative complications			
New pituitary insufficiency	5/50 (10%)	0/12 (0%)	0.57
SIADH or Diabetes insipidus	6/50 (12%)	1/12 (8.3%)	> 0.99

### Remission versus recurrence

35 patients with complete remission and 10 patients with tumor recurrence were compared.

No statistically significant differences between remission and recurrence rates were observed within different histological groups: densely granulated corticotrophic adenoma (28.6% vs. 30%, *p* > 0.99), sparsely granulated corticotrophic adenoma (68.6% vs. 70%, *p* > 0.99), and Crooke cell adenoma (2.9% vs. 0%, *p* > 0.99). Frequency of specimens with increased Ki67 expression ≥ 3% (15.2% vs. 20%, *p* = 0.66), p53 expression ≥ 2% (3.6% vs. 10%, *p* = 0.46) and mitosis per 10HPFs ≥ 2 (9.1% vs. 20%, *p* = 0.58) were not significantly different. Prevalence of cystic lesions (48.6% vs. 40%, *p* = 0.73) as well as incidence of patients with invasive growth of the tumor (80% vs. 100%, *p* = 0.32) were also not statistically distinct. The entire comparison is presented in Table 6.

**Table 6:** Remission versus recurrence after MTS.

Criteria	Patients with remission after MTS (n = 35)	Patients with recurrence after MTS (n = 10)	*p* value
Histological diagnosis			
Densely granulated corticotrophic adenoma	10/35 (28.6%)	3/10 (30%)	> 0.99
Sparsely granulated corticotrophic adenoma	24/35 (68.6%)	7/10 (70%)	> 0.99
Crooke cell adenoma	1/35 (2.9%)	0/10 (0%)	> 0.99
Histological markers			
Ki-67 ≥ 3%	5/33 (15.2%)	2/10 (20%)	0.66
P53 expression ≥ 2%	1/28 (3.6%)	1/10 (10%)	0.46
≥ 2 mitoses per 10 HPFs	3/33 (9.1%)	2/10 (20%)	0.58
Invasive growth	28/35 (80%)	10/10 (100%)	0.32
Cystic lesions	17/35 (48.6%)	4/10 (40%)	0.73

**Figure 2:**
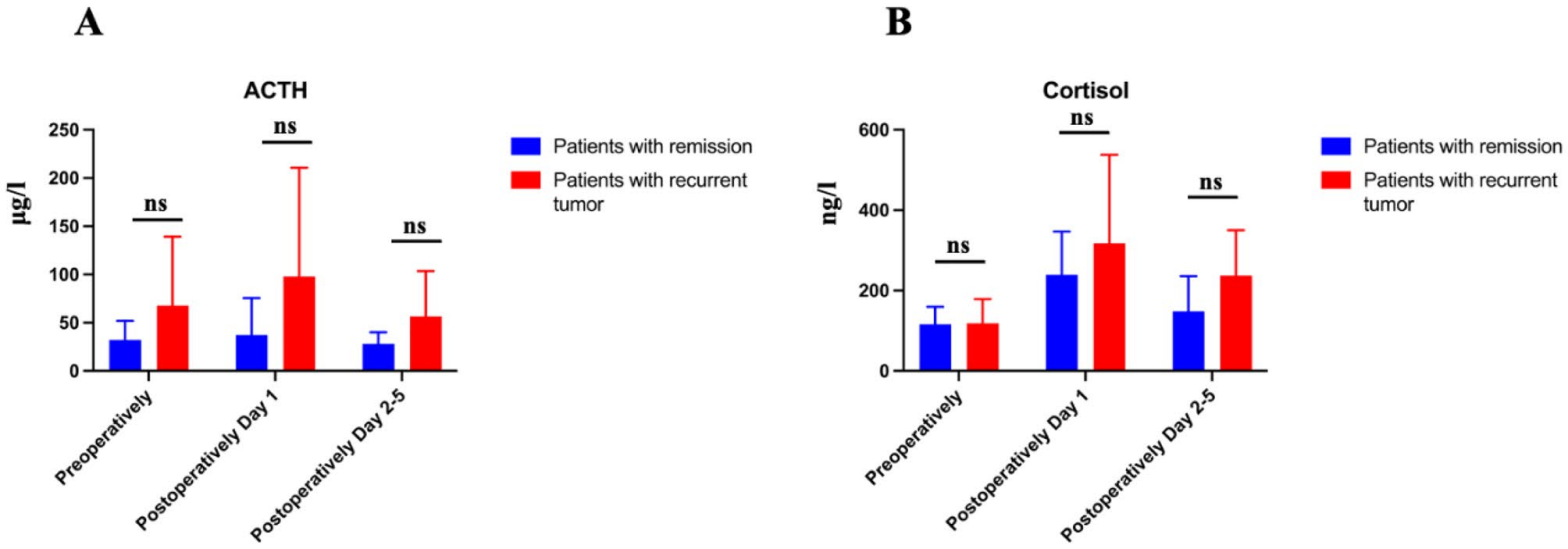
Cortisol and ACTH. Preoperative and postoperative (Day 1 and Day 2–5) ACTH (**A**) and cortisol (**B**) levels compared between patients with remission and recurrence after MTS. ACTH levels were higher by patients with recurrent tumors however without statistical significance(*p* = 0.18). No significant differences regarding cortisol levels were observed between both groups.

No statistically significant differences between two groups with respect to serum levels of cortisol and ACTH pre- and postoperatively were observed (Fig. 2).

## Discussion

### Surgical technique, complications and surgical features

Only a few case series of SCAs operated via (microscopic) transsphenoidal surgery can be found among the literature^10,25^.

In our study present one of the largest cohorts of SCAs operated via microscopic transsphenoidal surgery with extensive clinical and histological analyses. The most common complications were partial anterior pituitary insufficiency (8.1%) followed by diabetes insipidus (6.5%) and sinusitis (3.2%). Intraoperative bradycardia, which occurred in one case in our cohort may be the result of the trigemino-cardiac reflex (TCR)^26^. Other rare complications such as SIADH, CSF fistula, meningitis, abducens nerve palsy and postoperative bleeding have already been described. Remarkable is also a high incidence of intraoperative CSF space opening (32.3%), which may be result of large tumor size and invasive suprasellar growth.

The incidence of complete and partial pituitary insufficiency amounted preoperatively to 4.8% and 22.6%, respectively, and was lower than in other studies^10^. New postoperative hypopituitarism was restricted to five new partial insufficiencies (8.1%) and was lower than in other studies^25^. The new pituitary insufficiency may be explained by possible surgical damage of normal pituitary gland described by other authors^25^.

### Cystic versus non-cystic silent corticotrope adenoma

Cystic component is a very common finding among SCAs compared to other NFAs ^2,13,14^, but its clinical relevance has not yet been investigated. Previous studies revealed two different types of cystic components: microcysts (< 3 mm) and macrocysts within the tumors, and showed that the presence of multiple microcysts may be helpful to distinguish SCAs from other NFAs^2,14,15^. Our study presents the first clinical analysis of cystic versus non-cystic SCAs so far. Cystic tumors presented with characteristic clinical aspects like male predominance, higher risk of hyperprolactinemia as well as lower rates of cavernous sinus and sphenoid sinus invasion comparing to non-cystic lesions. Mean duration of first surgery was significantly shorter for cystic SCAs. Moreover preoperative intratumoral hemorrhage had 100% specificity and 60% sensitivity for densely granulated cystic SCAs. All these clinical hallmarks may suggest a novel subgroup of SCAs with distinct clinical and biological features, however further clinical and molecular investigations are required.

### Primary surgery versus repeat surgery

We performed the first comparison of index and repeat surgeries for SCAs.

First surgeries for NFAs reported in the literature presented with similar incidence of preoperative pituitary insufficiency, postoperative hypopituitarism, comparable mean time of surgery, higher incidence of diabetes insipidus and lower GTR rate compared with our study^27–29^. An alternative surgical method for NFAs remains the endoscopic transsphenoidal surgery (ETS) with higher GTR rate, similar incidence of diabetes insipidus/SIADH, postoperative hypopituitarism, intraoperative CSF space opening and longer surgical time comparing to MTS^27,30,31^.

In case of residual/recurrent pituitary adenoma, adjuvant radiotherapy or repeat surgery can be performed^32^. Repeat surgery is preferably conducted in symptomatic patients^32^. Results in the literature regarding repeat surgery, describing preoperative hypopituitarism (36%), no new postoperative pituitary insufficiency and GTR (26%) were comparable to the results of our study^33,34^. Endoscopic repeat surgery resulted in higher GTR (58.6%), lower incidence of new diabetes insipidus (4.9%), higher GTR (58.6%) and new pituitary insufficiency (9.7%) rate^32^.

### Remission versus recurrence

The predictors of recurrence in SCAs have already been analyzed by several authors and comprised Ki-67 proliferation index, mitotic index, p53 expression, cystic character, invasive growth, and GTR^12,35^. Cavernous sinus and suprasellar invasion along with STR were main factors associated with higher recurrence rate^12,25^. GTR significantly improved progression-free survival of SCAs^25^. Interestingly, patients with recurrence had less cystic tumors and higher baseline ACTH levels preoperatively, which was also observed in our study, however the results were not statistically significant^12^. Increased Ki67, mitotic rate or p53 expression were not connected to increased recurrence rate^12,35^, similar as has been shown in our cohort. The higher incidence of type 2 SCAs in our cohort has also been observed in other studies^9,12^.

### Study limitations

Our study is limited partly by the retrospective character of the analysis and a small number of patients, especially those with recurrence. Moreover, not all patients were available for follow-up analyses. p53 staining was not performed for all specimens. Based on retrospective analysis of MRI findings we could not differentiate between microcystic (< 3 mm) and macrocystic lesions within the tumors, which has been described in the literature^14,15^.

## Conclusions

In our study we present one of the largest available cohorts of patients after microscopic transsphenoidal surgery and first clinical analysis of cystic versus non-cystic SCAs so far. We also performed first comparison of index and repeat surgeries for these tumor entities.

Cystic tumors presented with characteristic clinical aspects like male predominance, higher risk of hyperprolactinemia as well as lower rates of cavernous sinus and sphenoid sinus invasion comparing to non-cystic lesions. Mean duration of first surgery was significantly shorter for cystic SCAs. Moreover preoperative intratumoral hemorrhage had 100% specificity and 60% sensitivity for densely granulated cystic SCAs. All these clinical hallmarks may suggest a novel subgroup of SCAs with distinct clinical and biological features, however further clinical and molecular investigations are required. No significant difference was found regarding preoperative pituitary insufficiency, intraoperative CSF space opening, new postoperative pituitary insufficiency or diabetes insipidus/SIADH, expression of histological markers such as Ki67, p53, mitotic rate and tumor recurrence were observed between cystic and non-cystic SCAs by patients who underwent first surgery.

Repeat surgery is associated with lower risk of GTR and higher incidence of preoperative pituitary insufficiency along with CSF space opening intraoperatively compared to primary surgery. The risk of new postoperative pituitary insufficiency and the incidence of diabetes insipidus/SIADH was similar between these groups. In our cohort no factors affecting tumor recurrence among the following could be detected: histological diagnosis, Ki67-prolifaration index, p53 expression, number of mitoses, invasive growth, or cystic lesions for SCAs.

## Data Availability

The datasets generated during and/or analysed during the current study are available from the corresponding author on reasonable request.

## Abbreviations

ACTH: Adrenocorticotropic hormone
CN: Cranial nerve
CSF: Cerebrospinal fluid
ETS: Endoscopic
FSH: Follicle-stimulating hormone
GH: Growth hormone
GTR: Gross total resection
HPF: High power field
LH: Luteinizing hormone
MRI: Magnetic resonance imaging
MTS: Microscopic transsphenoidal surgery
PH3: Phosphohistone-3
SIADH: Syndrome of inappropriate antidiuretic hormone secretion
SCA: Silent corticotrope adenoma
STR: Subtotal resection
TSH: Thyroid stimulating hormone
